# Basilar artery dolichosis is associated with a poor 90-day outcome in acute isolated pontine infarction

**DOI:** 10.1038/s41598-020-62873-4

**Published:** 2020-04-16

**Authors:** Shu-gang Cao, Xiaoxing Ni, Qian Wu, Jun He, Ping Cui, Tingting Ge, Yuancheng Li, Jian Wang, X. U. Wen’an, Mingwu Xia

**Affiliations:** 1 The Hefei Affiliated Hospital of Anhui Medical University, the Second People’s Hospital of Hefei, Department of Neurology, Hefei, Anhui 230011 P.R. China; 2 The Hefei Affiliated Hospital of Anhui Medical University, the Second People’s Hospital of Hefei, Department of Radiology, Hefei, Anhui 230011 P.R. China; 3 Department of Epidemiology, School of Public Health, Nanjing Medical University, Nanjing, Jiangsu 210000 P.R. China

**Keywords:** Cerebrovascular disorders, Stroke

## Abstract

Basilar artery (BA) dolichosis is not uncommon in patients with acute isolated pontine infarction. The effect of this abnormal BA geometrical form on the outcomes of pontine infarction has not been closely examined. This study aims to elucidate whether BA dolichosis contributes to a poor 90-day outcome in acute isolated pontine infarction. A total of 101 patients were enrolled with a median age of 65 years. The BA diameter (*p* = 0.026), basilar artery length (BAL) (*p* < 0.001), bending length (BL) (*p* < 0.001) and the proportion of BA bending (*p* < 0.001) were significantly higher in the BA dolichosis group. A poor outcome was closely associated with the baseline National Institute of Health Stroke Scale (NIHSS) score (*p* < 0.001), and BL (*p* = 0.042) as well as the proportions of BA dolichosis (*p* = 0.007) and BA bending (*p* = 0.010) in univariate analysis. Multivariate logistic regression analysis determined that BA dolichosis (adjusted OR = 4.724, 95% CI: 1.481~15.071, *p* = 0.009) and baseline NIHSS score (adjusted OR = 1.805, 95% CI: 1.296~2.513, *p* < 0.001) were independently associated with a poor outcome at 90 days. In conclusion, BA dolichosis may be a predictor of concern for a poor 90-day outcome in patients with acute isolated pontine infarction.

## Introduction

Vertebrobasilar dolichoectasia (VBD), first proposed in 1986 by Smoker *et al*.^1^ and then revised in 2005 by Caplan *et al*.^2^, is an uncommon and progressive vasculopathy that is characterized by the elongation and dilatation of the vertebrobasilar artery, including basilar artery dolichoectasia (BADE) and vertebral artery (VA) dolichoectasia. The overall population-based incidence rate of VBD is less than 0.05%^3^. Nevertheless, the incidence is higher in stroke patients^4–6^. A follow-up study of 156 patients with VBD revealed that the long-term outcome in VBD patients depended mainly on the severity of the condition and its evolutionary characteristics, and the progression of VBD posed a high risk of adverse events, especially stroke^7^. Nevertheless, vertebral artery dysplasia is very common, and the tortuosity of VAs varies widely, such as vascular bending, angling or folding, looping and spiraling, which will influence the accurate measurement of the VAs. Therefore, several studies have been conducted specifically regarding BADE^8,9^. Patients with BADE seem to be particularly susceptible to posterior circulation infarction^9,10^. Previous studies have shown that the infarct sites of BADE patients are mainly in the pons, especially in the paramedian pontine^5,9–11^.

Furthermore, in Chinese population, few patients were diagnosed with BA ectasia or BADE based on the current diagnostic criteria for BADE^12,13^. But there are many cases with a simply elongated BA that cannot be classified as BADE. The effect of this abnormal BA geometrical form on the outcomes of stroke has not been well examined. BA dolichosis is not uncommon especially in patients with pontine infarction. Although several previous clinical and/or imaging studies have been conducted specifically for BADE in pontine infarction, there are few studies regarding the relationship between BA dolichosis and clinical outcomes in acute pontine infarction. Therefore, the purpose of this study is to elucidate whether BA dolichosis contributes to a poor outcome 90 days after onset in patients with acute isolated pontine infarction.

## Results

### Clinical and demographic data of the study

**Figure 1:**
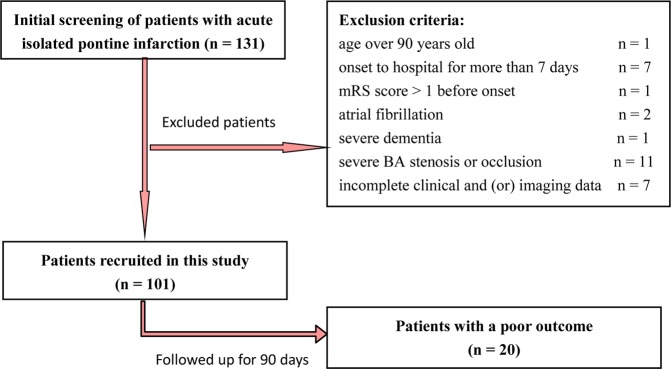
The schematic diagram shows the selection of patients with acute isolated pontine infarction.

**Figure 2:**
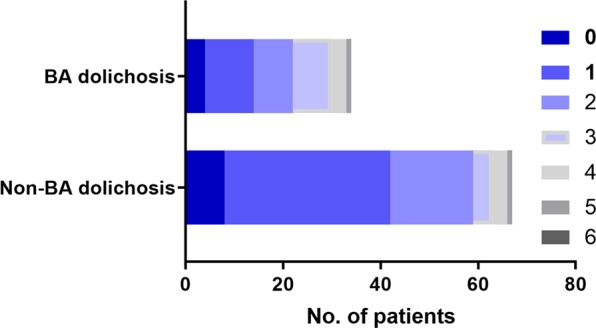
The mRS score distribution between the BA dolichosis group and the non-BA dolichosis group.

In total, 131 patients with acute isolated pontine infarction were admitted during the study period between July 2015 and June 2018. Among these patients, 30 were excluded according to the exclusion criteria. There was no loss of follow-up or death during the 90-day follow-up. Therefore, 101 patients were finally enrolled in the present study (Fig. 1). All patients were Chinese Hans. Among the 101 patients, 60 (59.4%) were male and 41 (40.6%) were female with an age of 65 (20) (years, median (M) and interquartile range (IQR)). The infarct sites of 80 (79.2%) were located in the paramedian pontine area, 4 (4.0%) were in the anterior lateral pontine area, 9 (8.9%) were in the lateral pontine area and 8 (7.9%) were in multiple infarct areas. Patients with simple BA dolichosis accounted for 32.7% (33 cases), but no patients had simple BA ectasia. BADE patients accounted for only 1.0% (1 case). Of the 101 patients, 34 (33.7%) patients were divided into the BA dolichosis group, and the other 67 (66.3%) patients were divided into the non-BA dolichosis group. There were 81 (80.2%) patients with good outcomes versus 20 (19.8%) patients with a poor outcome at 90 days. The characteristics of the study population divided by group are summarized in Fig. 2 and Supplementary Table 1.

### Geometrical properties between the BA dolichosis and non-BA dolichosis groups

**Figure 3:**
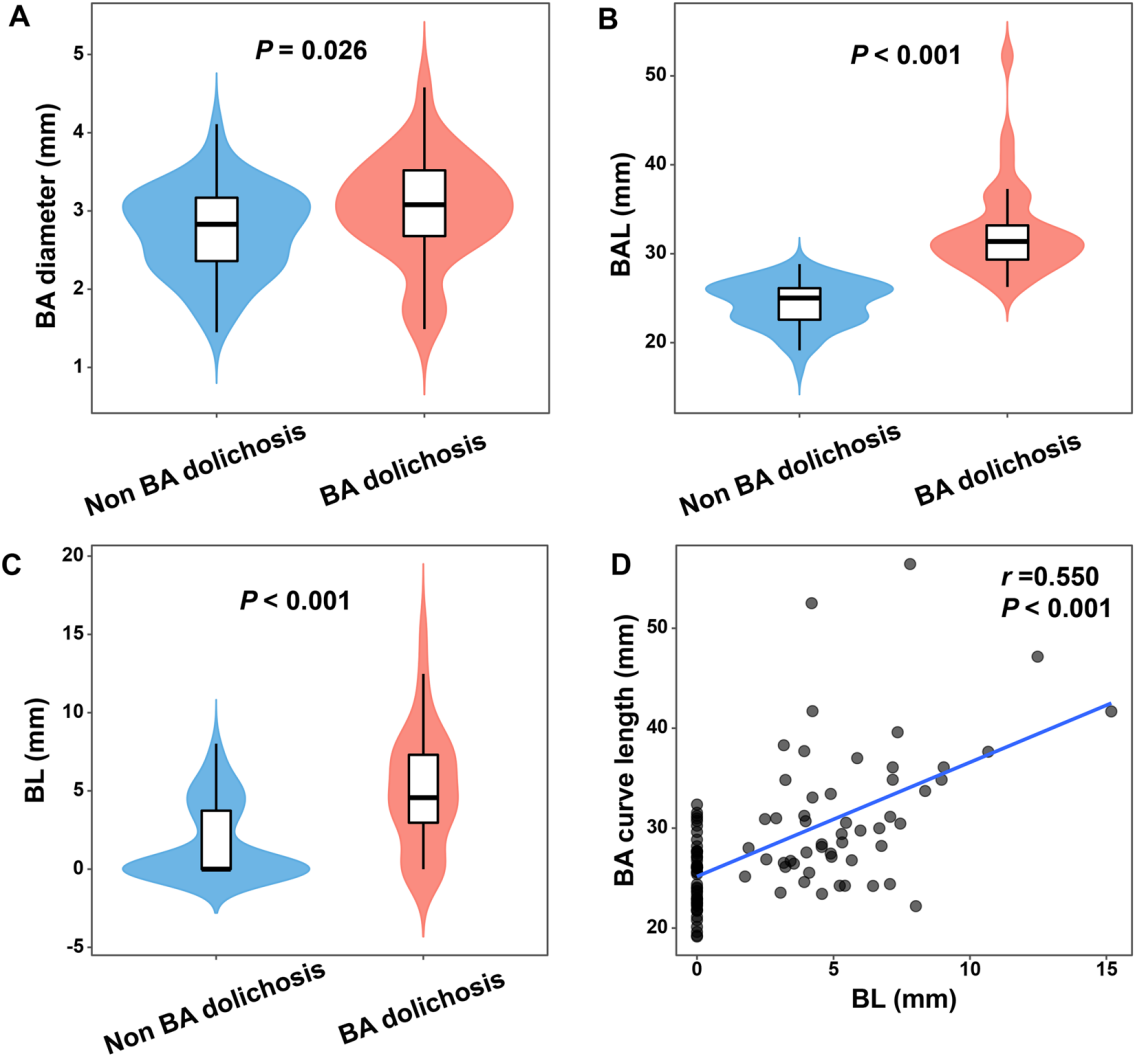
Comparison of BA diameter, basilar artery length (BAL) and bending length (BL) between the BA dolichosis and the non-BA dolichosis group (**A–C**). Positive correlation between BA curve length and BL (n = 101) (**D**).

Compared with the non-BA dolichosis group, some geometrical properties of the BA were significantly greater in patients with BA dolichosis, including BA diameter (*p* = 0.026), basilar artery length (BAL) (*p* < 0.001), bending length (BL) (*p* < 0.001), and the proportion of BA bending (*p* < 0.001). However, there were no significant differences between the two groups with regard to the proportion of BADE, BA stenosis and BA hypoplasia (BAH), as well as infarct size (Fig. 3 and Table 1).

**Table 1:** Vascular geometrical properties and infarct size between the BA dolichosis group and the non-BA dolichosis group.

Variables	BA dolichosis (n = 34)	non-BA dolichosis (n = 67)	*p value*
BA diameter (mm)	3.02 ± 0.67	2.74 ± 0.56	0.026
**Length of BA**			
BAL (mm)	31.38 (29.21, 33.79)	25.02 (22.50, 26.13)	<0.001
BL (mm)	4.57 (2.80, 7.38)	0 (0, 3.93)	<0.001
BA bending, n (%)	28(82.4)	25(37.3)	<0.001
BADE, n (%)	1(2.9)	0(0)	0.728*
BAH, n (%)	4(11.8)	7(10.4)	1.000
BA stenosis, n (%)	5(14.7)	6(9.0)	0.590
Infarct size (mm)	15.47 ± 5.38	14.46 ± 4.67	0.335

BA basilar artery, BAL basilar artery length, BL bending length, BADE basilar artery dolichoectasia, BAH basilar artery hypoplasia. *Fisher’s Exact Test.

### Correlation analysis between BL and BA curve length

Spearman correlation analyses showed a positive correlation between BA curve length and BL (correlation coefficient r = 0.550, *p* < 0.001) which is displayed in Fig. 3.

### Univariate and multivariate logistic regression analysis of risk factors associated with a poor 90-day outcome

**Figure 4:**
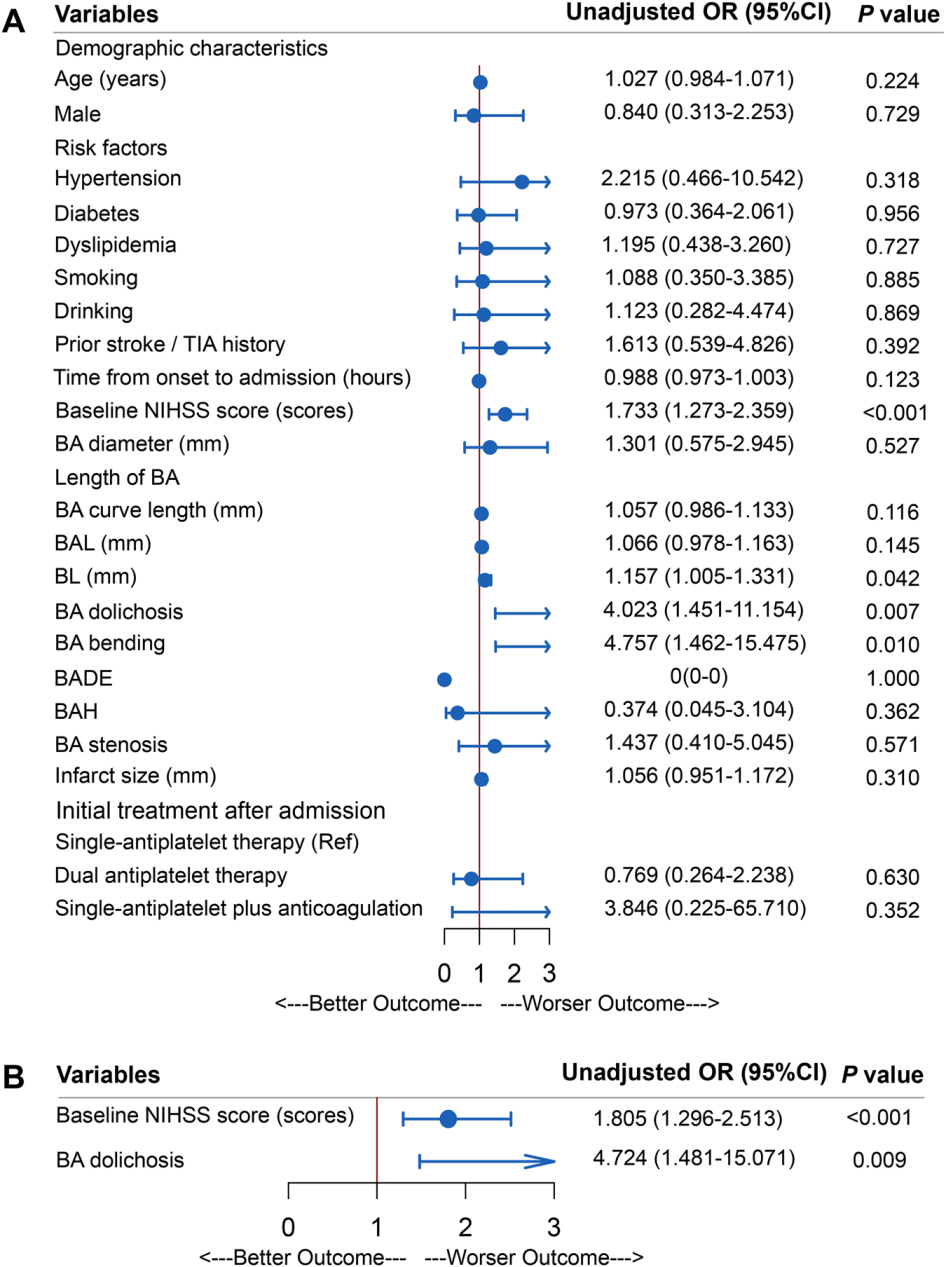
Univariate and multivariate logistic regression analysis of risk factors associated with a poor 90-day outcome in acute isolated pontine infarction.

Baseline National Institute of Health Sstroke Sscale (NIHSS) score (*p* < 0.001), BL (*p* = 0.042), and the proportion of BA dolichosis (*p* = 0.007) and BA bending (*p* = 0.010) were significantly greater in patients with a poor outcome in univariate logistic regression analysis. Multivariate logistic regression analysis showed that the baseline NIHSS score (OR = 1.805, 95% CI: 1.296–2.513, *p* < 0.001) and BA dolichosis (OR = 4.724, 95% CI: 1.481∼15.071, *p* = 0.009) were closely associated with a poor 90-day outcome after adjusting for variables with potential associations. However, other factors showed no association with a poor outcome (Fig. 4).

## Discussion

In this study, we found that BA dolichosis was highly associated with a poor outcome at 90 days in patients with acute isolated pontine infarction. In addition, the baseline NIHSS score was also closely associated with a poor outcome. Therefore, BA dolichosis may represent a readily available prognostic predictor for acute isolated pontine infarction.

In recent years, several studies have been conducted specifically for BADE because of the great variability of the properties of the VAs^8,9^. A recent study showed that in community-dwelling older adults over 60 years old, the proportion of BA ectasia and/or dolichosis was as high as 13.6%^8^. Kwon *et al*. revealed that the proportion of BADE in patients with acute pontine infarction reached up to 18.8%, and BADE was associated with paramedian pontine infarction^5^. In the present study, the proportion of BA dolichosis including 1 patient with BA dolichosis and ectasia reached 33.7% in patients with acute isolated pontine infarction. Among them, patients with simple BA dolichosis accounted for 32.7%, but no patients had simple BA ectasia. However, surprisingly, BADE patients accounted for only 1.0%, which might be the result of different diagnostic criteria, study populations, and ethnicities compared to other studies. Currently, the diagnostic criteria for BADE used in China are derived from non-Asian populations^13^. Deng *et al*.^12^ previously proposed diagnostic criteria for BA ectasia for Chinese population; that is, males with a BA diameter >4.2 mm and females with a BA diameter >4.0 mm should be diagnosed with BA ectasia. The possible racial and gender differences in BA diameter may explain why there were so few patients with simple BA ectasia or BADE in this study.

The present study first compared BA geometrical properties between patients with and without BA dolichosis, which showed that the BA diameter of patients in the BA dolichosis group was larger than that in the non-BA dolichosis group, and the proportion of BA bending in the BA dolichosis group was also higher than that of the non-BA dolichosis group, suggesting that patients with BA dolichosis were more likely to have BA bending or ectasia (1 patient with BA dolichosis also had BA ectasia). Spearman correlation analysis further confirmed that BA curve length was positively correlated with BL. These BA features are related not only to the genetic development of BA itself^14,15^, but also to VA dominance or dysplasia^15^. In light of the previous vascular remodeling theory, it is presumed that the VAs are typically asymmetric, with the BA gradually curving in the opposite direction from the larger VA, thus aggravating BA dilatation or elongation with secondary perforating artery diseases and subsequent hemodynamic changes, which might eventually lead to thrombosis, microembolization and brainstem compression^9,16,17^.

Many patients with an elongated BA cannot be classified as BADE. There is much less information about the effects of this abnormal BA geometrical form on outcome. The present study showed that patients with acute isolated pontine infarction complicated with BA dolichosis had higher baseline NIHSS scores and poorer outcomes at 90 days. The infarct sites of BADE patients are mainly located in the pons, particularly in the paramedian pontine area^10,11,18^, which is also the pathway of the corticospinal tract. In this study, the infarct sites of up to 80 (79.2%) patients were located in the paramedian pontine in accordance with previous views. Although there was no statistically significant difference in infarct size between the BA dolichosis group and the non-BA dolichosis group, the patients in the BA dolichosis group had a larger infarct size, and more fiber bundles of the pons might be involved, especially the corticospinal tract in the basal pontine. Correspondingly, the baseline NIHSS score would be higher and the outcome would be relatively worse, thereby more profoundly supporting this inference. Although BA ectasia was not an independent predictor of a poor outcome, the BA diameter of the poor outcome group was greater than that of the good outcome group, which might also play a role in the pathogenesis and result in a poorer outcome.

The pathogenesis of pontine infarction as a consequence of BA dolichosis may be invovled in hemodynamic abnormalities, *in situ* thrombosis or embolism, perforating arterial disease due to mechanical compression or traction, or multiple mechanisms. Taking previous findings into account, it appears that several factors may account for the causal association between BA dolichosis and pontine infarction. First, BA dolichosis is often combined with BA bending or ectasia, all of which can lead to hemodynamic changes, make the vessel wall prone to atherosclerotic changes and result in ischemia in the perforating branch of the BA^19^. Taking BA bending as an example, the blood flow impact zone, low shear stress zone and slow flow zone would appear after the bent artery, which might result in injury to the local vascular intima more easily and aggravation of atherosclerotic angiopathy^19^. Blood flow stagnation and turbulence caused by the decrease of blood flow velocity at the site of BA dilatation can lead to repeated thrombosis, which may occlude large blood vessels such as the BA, block the opening of perforator arteries or break apart into microemboli with subsequent microvascular obstruction^20^. Second, whether the BA is elongated, bent or dilated, the subsequent compression and/or stretching of the perforating artery could possibly result in vasospasm and increase atherosclerotic changes in the perforating arteries^5,9^. Furthermore, the mechanical stretching of the BA bending and angulation caused by BA dolichosis could deform the branch of the perforating artery and reduce blood perfusion. The BA perforating branch is most likely to be stretched and twisted, leading to infarction of the BA^20^. Studies have also revealed that infarcts are mostly located on the opposite side of the BA offset position and may be associated with the perforating arterial lesions caused by such mechanical traction^10,21^.

To the best of our knowledge, this study was the first observational prospective cohort study with high-quality imaging data measurement and follow-up to investigate the relationship between BA dolichosis and the short-term outcome in acute isolated pontine infarction. This study also has some limitations. First, the study population is a small sample of the Han population, and whether it can be applied to other populations still needs to be further confirmed by a large sample and multicenter study. Second, the geometric parameters of the BA were mainly obtained via MRA (currently widely used in other studies), so the accuracy of measurement is relatively low compared with CT angiography (CTA) or digital subtraction angiography (DSA). In addition, this study did not evaluate the geometric parameters of the VAs and the hemodynamics of the vertebrobasilar artery, which may be helpful in elucidating the mechanism of BA dolichosis susceptible to pontine infarction. Finally, this study only analyzed the short-term prognostic effects of BA dolichosis on acute isolated pontine infarction, while the long-term prognostic effects will be further investigated later through longer follow-up.

## Conclusion

BA dolichosis was an important imaging marker that carries prognostic value with regards to a poor outcome for acute isolated pontine infarction and could be a potential point of concern in clinical treatment and outcome assessment.

## Methods

### Subjects

This prospective cohort study collected consecutive stroke patients with pontine infarction who were admitted from July 2015 to June 2018 in the Affiliated Hefei Hospital of Anhui Medical University. All patients were registered in Anhui Stroke Network Registry.

Patients were included in the analysis if they were aged 18 to 90 years old, admitted within 7 days after onset and diagnosed with acute isolated pontine infarction by diffusion weighted imaging (DWI). Patients were excluded according to the following conditions: if they were aged over 90 years old, were admitted with time from onset to hospital of more than 7 days, had an mRS score >1 before this onset in case of recurrent cerebral infarction, had a previous history of atrial fibrillation or newly diagnosed atrial fibrillation, had severe dementia, had segmental thickening of the BA or BA aneurysms, had evidence of haemodynamically severe stenosis (≥70%) or occlusion of the BA on initial MRA affecting data measurements, or had incomplete clinical or imaging information.

### Demographic characteristics and risk factors

Detailed demographic information (including gender and age), personal history (including current smoking or drinking) and medical history records (hypertension, diabetes, dyslipidemia, prior stroke or transient ischemic attack (TIA), etc.) indicating their vascular risk factors were systematically acquired. All patients with vascular risk factors had been previously or newly diagnosed as such and/or were already taking medications for these conditions.

### Assessment of the severity of pontine infarction

The severity of acute pontine infarction was evaluated according to the National Institute of Health Sstroke Sscale (NIHSS) score, which is widely used to evaluate the severity of acute ischemic stroke. The baseline NIHSS score was determined by a trained neurologist on admission.

### Imaging analysis

All patients received magnetic resonance imaging (MRI) scanning with a 1.5 Tesla MRI scanner (Siemens Healthineers, Model: Avanto I class, Germany). Scanning was performed with conventional T_1_-weighted imaging (T_1_WI), T_2_-weighted imaging (T_2_WI), fluid attenuated inversion recovery (FLAIR), DWI and apparent diffusion coefficient (ADC) sequences. The DWI sequence was performed with the following parameters: repetition time/echo time, 3400/102 ms; slice thickness, 5 mm; dispersion mode: 3-scan trace; b value, 0–1000. Three-dimensional time-of-flight (3-D TOF) magnetic resonance angiography (MRA) was acquired in the axial plane by using the following parameters: repetition time 25 ms, echo time 7 ms, and section thickness 0.6 mm. The geometrical parameters of the BA were analyzed by syngo 3-D VesselView.

### BA features

**Figure 5:**
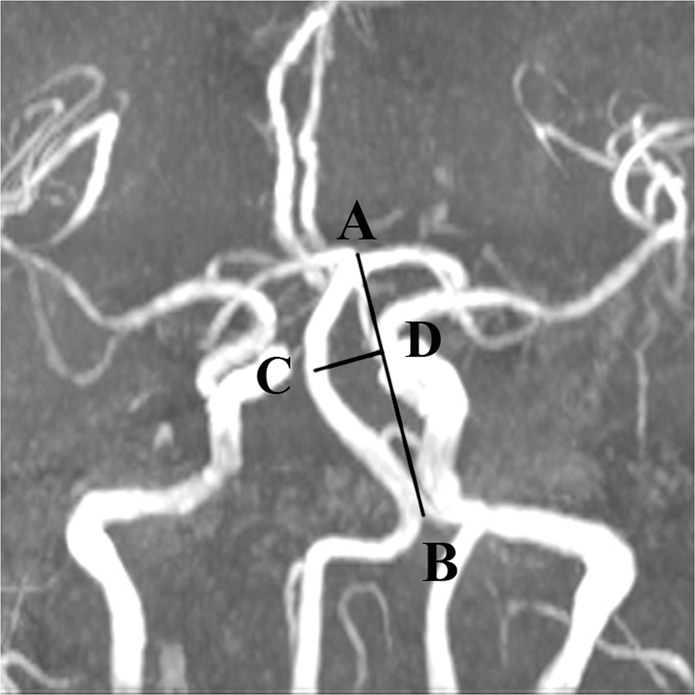
The sites of measurement of the BA via magnetic resonance angiography (MRA)^16^. AB is basilar artery length (BAL), and CD is bending length (BL).

From the 3-D TOF MRA, BA stenosis was measured based on the North American symptomatic carotid endarterectomy test^22^. The rate of BA stenosis ≥50% was diagnosed as BA stenosis. The shape of the BA was measured according to the standard established by Hong *et al*.^23^ and was classified into 4 groups: (1) straight (no bending); (2) C-shaped (toward the right side of the body); (3) inverted C-shaped (toward the left side); and (4) S-shaped (multiple bending similar to S-type). The measurement of the geometrical properties was taken from the main trunk of the BA. Four parameters were measured from the BA: (1) maximum diameter, (2) curve length, (3) BAL^16^, which is a linear distance from the confluence point of the bilateral VAs to the initial point of the BA division into the bilateral posterior cerebral arteries, and (4) BL^16^. The BL criteria are as follows: for the C-shaped or inverted C-shaped BA, BL was defined as the vertical distance from the bending point of the BA to the standard line, namely, the BAL (Fig. 5). For the S-shaped BA, BL was defined as the length difference between the two lateral deviations on both sides. These parameters were continuously measured twice by two neurologists (J.W. and Q.W.) at the same time, and the average of each was taken as the final value. For the S-shaped BA, the bending direction was decided independently by these two neurologists. If any discrepancies were encountered, a radiologist with 10-year experience (P.C.) was consulted to resolve the issue. The radiologist and two neurologists were blinded to the clinical and outcome information.

### BADE and BA hypoplasia criteria

BADE was diagnosed on the basis of the criteria established by Ubogu^13^. According to the criteria, BA ectasia was defined as an arterial diameter >4.5 mm at any location along its course. BA curve length >29.5 mm or BL >10 mm was considered as BA dolichosis. Cases meeting the above two criteria simultaneously were diagnosed with BADE. Segments with focal disease (e.g., basilar artery aneurysm) were not included.

BAH was defined as a continuous diameter reduction throughout the artery and a BA diameter <2.0 mm in any location along its course^24^.

### The characteristics of pontine infarction

The pontine infarction sites are divided into the paramedian region, the anterior lateral region, the lateral region and the multiple infarct areas (i.e., the infarction in the border zones of two adjacent perforating arterial territories) according to the vascular anatomy of the pons. The maximum diameter at the largest infarct level on DWI was taken as the infarct size.

### Evaluation of 90-day outcome

The 90-day modified Rankin Scale (mRS, a seven-level ordered categorical scale, where 0 = independent and 6 = dead) score^25^ was used to measure the neurological functional recovery of patients with acute pontine infarction. The patients were divided into two groups according to the clinical outcome: the good outcome group (mRS: 0~2) and the poor outcome group (mRS: 3~6). The mRS was acquired from each patient through telephone or outpatient follow-up 90 days after onset. The follow-up was performed by a trained neurologist (S.C.) blinded to all imaging manifestations.

### Statistical analysis

All statistical analysis were performed using SPSS for Windows (SPSS version 18.0). Continuous variables were tested for normality using the Kolmogorov-Smirnov test. Normally distributed variables are expressed as the mean ± standard deviation (mean ± SD), while nonnormally distributed variables are shown as the median (M) and interquartile range (IQR). Categorical variables are expressed as absolute numbers and percentages (%). Differences in continuous variables between groups were assessed by Student’s *t* test (normally distributed) or Mann-Whitney U test (nonnormally distributed). Differences in categorical variable distributions between groups were assessed by the *χ*^2^ test or Fisher’s exact test as appropriate.

First, the vascular geometries of the BA and the infarct size were compared between patients with and without BA dolichosis. Second, univariate and multivariate logistic regression were performed to verify the factors independently associated with 90-day outcome after onset. Epidemiological information, baseline NIHSS score, infarct size and the BA geometrical properties of patients with poor outcomes were compared with those with good outcomes. Variables with a potential association with a 90-day poor outcome (*p* < 0.1) from univariate analysis were used for multivariate analysis. Binary logistic regression (forward stepwise) was used to verify factors independently associated with a 90-day outcome. Odds ratio (OR) and 95% confidence interval (CI) were subsequently calculated. Potential relationships among variables were tested by Spearman correlation analysis, and the correlation coefficient was expressed by *r*. All tests used a two-sided *p* value of 0.05 as a threshold for significance. All plots were draw in R software (version 3.6.1), using ggplot2 (version 3.2.1) and forestplot (version 1.9) packages. A Bland-Altman plot was used to analyze the agreement between the 2 readers (Supplementary Figs. 1–3).

### Ethical approval and consent to participate

This study was approved by the Institutional Review Board of the Affiliated Hefei Hospital of Anhui Medical University and therefore was performed in accordance with the ethical standards laid down in the 1964 Declaration of Helsinki and its later amendments. All patients gave their informed consent prior to their inclusion in the study. Informed consent was obtained directly from the patient or from a family member or other legal guardian. All patients or their key relations were informed of the purpose of the study.

## Supplementary information

Supplementary information.

## Data Availability

All data generated or analyzed for this study are included in this published article (and its Supplementary Information files). The datasets generated during and/or analyzed during the current study are available from the corresponding author on reasonable request.
