# Supplementary material for: Basilar artery dolichosis is associated with a poor 90-day outcome in acute isolated pontine infarction
Source: Sci Rep. 2020 Apr 16;10:6557. doi: 10.1038/s41598-020-62873-4 (PMC7162970; doi:10.1038/s41598-020-62873-4)
Supplement: Supplementary file 1 — Supplementary information. [file 41598_2020_62873_MOESM1_ESM.pdf]

Basilar artery dolichosis is associated with a poor 90-day outcome in acute isolated pontine infarction

Shu-gang Cao[1,+], Xiaoxing Ni[1,+], Qian Wu[1] , Jun He[1], Ping Cui[2], Tingting Ge[1], Yuancheng Li[3], Jian Wang[1], Wen'an XU[1], Mingwu Xia[1,*]

[1] the Hefei Affiliated Hospital of Anhui Medical University, the Second People's Hospital of Hefei, Department of Neurology, Hefei, Anhui 230011, P.R. China

[2] the Hefei Affiliated Hospital of Anhui Medical University, the Second People's Hospital of Hefei, Department of Radiology, Hefei, Anhui 230011, P.R. China

[3] Department of Epidemiology, School of Public Health, Nanjing Medical University, Nanjing, Jiangsu 210000, P.R. China

[*]Corresponding author: Mingwu Xia, Email: xiamingwu1965@163.com

[+]these authors contributed equally to this work

Supplementary table 1 Comparison of associated factors in patients with different 90-day outcomes.

Variables	90-day outcome		*p value*
	poor (n = 20)	good (n = 81)	
**Demographic data**			
Age (years)	70.5 (60.3, 78.8)	63.0 (56.5, 76.0)	0.218
Male, n (%)	11 (55.0)	48 (59.3)	0.729
**Risk factors**			
Hypertension, n (%)	18 (90.0)	65 (80.2)	0.487
Diabetes, n (%)	9 (45.0)	37 (45.7)	0.956
Dyslipidemia, n (%)	8 (40.0)	29 (35.8)	0.727
Smoking, n (%)	5 (25.0)	19 (23.5)	1.000
Drinking, n (%)	3 (15.0)	11 (13.6)	1.000
Prior stroke / TIA history, n (%)	6 (30.0)	17 (21.0)	0.573
**Time from onset to admission** (hours)	14.5 (7.0, 50.0)	45.0 (18.0, 69.5)	0.218
**Baseline NIHSS score** (scores)	7 (6, 8)	5 (3, 6)	<0.001
**BA diameter** (mm)	2.91±0.44	2.82±0.65	0.531
**Length of BA**			
Curve length (mm)	30.61 (26.81, 34.54)	26.44 (23.57, 30.10)	0.012
BAL (mm)	28.39 (25.61, 31.44)	26.04 (22.95, 28.64)	0.039
BL (mm)	4.12 (2.97, 6.97)	0 (0, 4.74)	0.011
**BA dolichosis**, n (%)	12 (60.0)	22 (27.2)	0.005
**BA bending**, n (%)	16 (80.0)	37 (46.7)	0.006
**BADE**, n (%)	0 (0)	1 (1.2)	1.000*
**BAH**, n (%)	1 (5.0)	10 (12.3)	0.587
**BA stenosis**, n (%)	4 (20)	12 (14.8)	0.821
**Infarct size** (mm)	15.80 ±4.61	14.55 ±4.98	0.312
**Initial treatment after admission**			
Dual antiplatelet therapy, n (%)	6 (30.0)	30 (37.0)	0.558
Single-antiplatelet therapy, n (%)	13 (65.0)	50 (61.8)	
Single-antiplatelet plus anticoagulation, n (%)	1 (5.0)	1 (1.2)	

TIA transient ischemic attack, BA basilar artery, BAL basilar artery length, BL bending length, BADE basilar artery dolichoectasia, BAH basilar artery hypoplasia. * Fisher's Exact Test

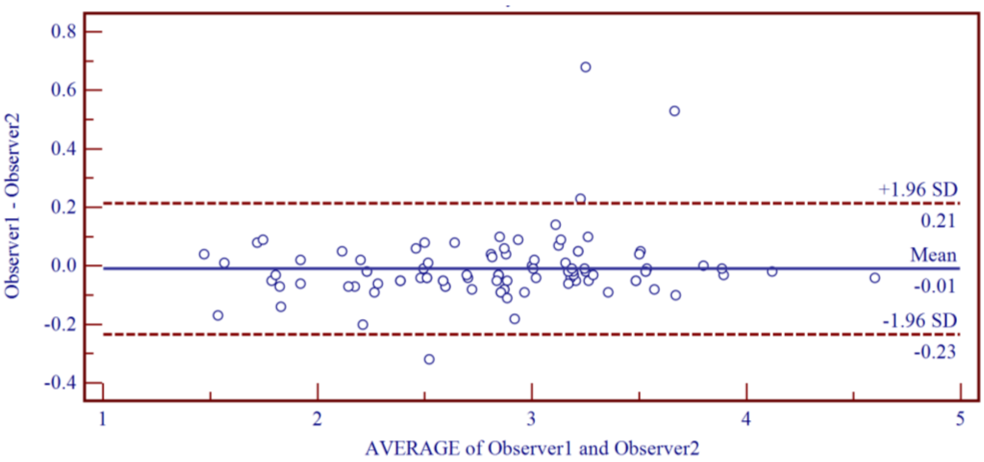

Supplementary Figure 1. Bland-Altman plot of BA diameter of the senior and junior readers.

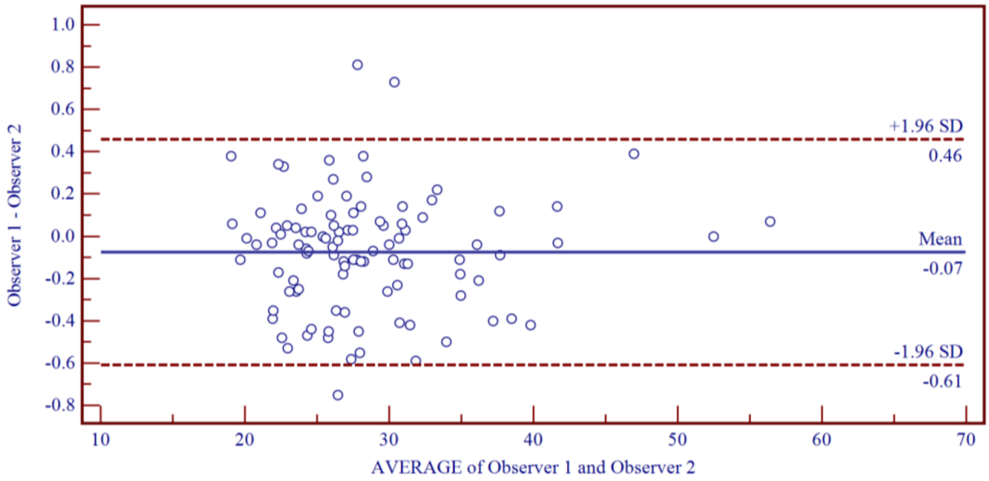

Supplementary Figure 2. Bland-Altman plot of BA curve length of the senior and junior readers.

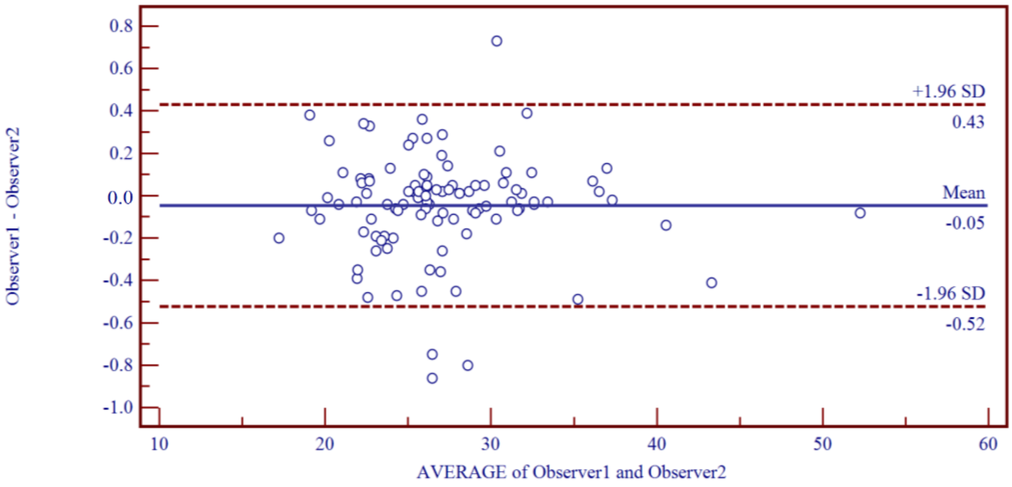

Supplementary Figure 3. Bland-Altman plot of basilar artery length (BAL) of the senior and junior readers.
